# The efficiency of quantum teleportation with three-qubit entangled state in a noisy environment

**DOI:** 10.1038/s41598-023-30561-8

**Published:** 2023-03-07

**Authors:** Chang-Yue Zhang, Zhu-Jun Zheng, Zhao-Bing Fan, Hai-Tao Ma

**Affiliations:** 1 Department of Mathematics, South China University of Technology, Guangzhou, 510641 People’s Republic of China; 2 Laboratory of Quantum Science and Engineering, South China University of Technology, Guangzhou, 510641 People’s Republic of China; 3 College of Mathematics Science, Harbin Engineering University, Harbin, 15001 People’s Republic of China

**Keywords:** Applied mathematics, Computational science

## Abstract

Quantum teleportation plays a significant role in the field of quantum communication. This paper investigates quantum teleportation through a noisy environment by using GHZ state and non-standard *W* state as quantum channels. We analyze the efficiency of quantum teleportation by solving analytically a master equation in Lindblad form. Following the quantum teleportation protocol, we obtain the fidelity of quantum teleportation as a function of evolution time. The calculation results show that the teleportation fidelity using non-standard *W* is higher in comparison to GHZ state at the same evolution time. Moreover, we consider the efficiency of teleportation with weak measurements and reverse quantum measurement under amplitude damping noise. Our analysis suggests that the teleportation fidelity using non-standard *W* is also more robust to noise than GHZ state in the same conditions. Interestingly, we found that weak measurement and its reverse operation have no positive effect on the efficiency of quantum teleportation by using GHZ and non-standard *W* state in the amplitude damping noise environment. In addition, we also demonstrate the efficiency of quantum teleportation can be improved by making minor modifications to the protocol.

## Introduction

Quantum entanglement is shown to be an efficient resource for many potential applications that cannot be achieved using classical resources, such as quantum teleportation^1^, quantum cryptography^2^, quantum computing^3^, quantum dense coding^4^, etc. Quantum teleportation (QT) as a typical application of quantum entanglement, which is proposed by Bennett et al.^1^ in 1993 and demonstrated experimentally a few years later by Bouwmeester et al.^5^. In the original teleportation scheme^1^, Alice transmits an unknown quantum state to Bob by using a maximally entangled state as quantum channel. In 1998, Karlsson et al.^6^ proposed a protocol that teleport a quantum state by using three-particle entanglement, which has a high degree of security. Based on it, Agrawal et al.^7^ investigated a class of *W* state that can be used for perfect teleportation. And compared to the GHZ state, the *W* state can still be used as a resource even after particle loss. Thereafter, teleportation using other multi-particle entangled states has also been considered^8–10^. Because of the properties of quantum entanglement^11–14^, QT brings some new capabilities that are probably impossible to attain in any classical communication.

In reality, however, quantum entanglement is subjected to decoherence due to the inevitable interaction of entangled qubits with the environment^15–18^. In QT protocol, the maximally entangled state becomes mixed state due to the inevitable interactions with the environments during entanglement distribution. Hence, the dynamics of quantum entanglement under a noisy environment always affects QT. Naturally, ones need to consider the influence of entanglement evolution in a noisy environment on QT^19–23^. In 2015, Fortes et al.^21^ investigated the efficiency of QT in the condition that qubits are subjected to noise. Then, the efficiencies of partially entangled states in three-qubit classes under real conditions are analyzed in Re^22^. Fonseca^23^ studied the protocol of qudit teleportation using quantum systems subjected to several kinds of noise for arbitrary dimensionality *d*. Moreover, the minimum assured fidelity (MASFI) of QT has been studied, which is the best measure of the quality of quantum teleportation protocol when the quantum channel is imperfect^24–26^.

We noted that little work has focused on the relationship between teleportation efficiency and evolution time in a noisy environment. Nevertheless, lots of studies on the dynamics of quantum entanglement focus on the time evolution of the quantum system^27–32^. So, we investigate the variation of fidelity by evolution time when three-qubit entangled state is used as quantum channels for QT under a noisy environment. We focus on the amplitude damping noise, which is a prototype model of a dissipating interaction between the quantum system and its zero-temperature environment^15^. Furthermore, some previous works employed the technique of weak measurement (WK) and reverse quantum measurement (RM) in order to enable the preservation of quantum entanglement^33–38^. The fidelity of quantum teleportation with WK and RM operations is also studied here.

To begin with, we describe the standard quantum teleportation in a noisy environment and give a framework for calculating the fidelity of teleportation. Based on Re^7^, we concern two typical states of three-qubit entangled state as quantum channels, GHZ state and non-standard *W* state, which are affected by amplitude damping noise. Subsequently, we calculate the fidelities of the final output states by solving Lindblad’s master equation in the amplitude damping noise environment. And the relationship among the efficiency of teleportation, decoherence time and initial states is established. We also make a comparison of fidelities of QT between GHZ state and non-standard *W* under the help of WM and RM. We find that the fidelity of teleportation cannot be increased after using WM and RM to protect GHZ and non-standard *W* states for the given protocol. Combined with the analysis above, we demonstrate the efficiency of quantum teleportation can be improved by changing the participant who prepares the initial state under amplitude damping, bit flip, bit-phase flip and phase flip noises.

## Results

### Mathematical description of quantum teleportation in noisy environments

To start, we consider the procedure of QT using an arbitrary three-qubit to transmit quantum information under noisy environments. Due to the limitation of entanglement distribution distance, we describe the protocol in the situation where Dave is honesty and located in the middle of Alice and Bob. To increasing transmission distance, in what follows we give the mathematical description for a general QT protocol that qubits are subjected to noise, which is characterized by Lindblad master equation.

Without loss of generality, suppose that Alice wants to transmit an unknown state $|\varphi \rangle _a$ to Bob, which is

1 $$\begin{aligned} |\varphi \rangle _a=\alpha |0\rangle _a+\beta |1\rangle _a, \end{aligned}$$

where $|\alpha |^2+|\beta |^2=1.$ Its density matrix is

2 $$\begin{aligned} \rho _{in}=|\varphi \rangle _a\langle \varphi |_a. \end{aligned}$$

*Step 1*: Consider that Dave prepares a three qubit entangled state $|\phi \rangle _{123}$, whose density matrix formalism is
  3 $$\begin{aligned} \hat{\rho }=|\phi \rangle _{123}\langle \phi |_{123}. \end{aligned}$$
  Therefore, the initial total state describing the whole system before any quantum operation is implemented is given by
  4 $$\begin{aligned} \rho =\rho _{in}\otimes \hat{\rho }. \end{aligned}$$
*Step 2*: Dave sends particles 1 and 2 to Alice, particle 3 to Bob, respectively. In practical, the particles to be sent are affected by the noises. 
  Here, we adopt the master equation approach that describes quantum noise in continuous time using differential equations. For an open system, which is affected by environmental influences and other factors can be characterized by Lindblad master equation^15,18^,
  5 $$\begin{aligned} \frac{\partial \rho }{\partial t}=-i[H_s,\rho ]+\sum _{i, \alpha }\left( L_{i, \alpha }\rho L_{i, \alpha }^\dagger -\frac{1}{2}\left\{ L_{i, \alpha }^\dagger L_{i, \alpha },\rho \right\} \right) , \end{aligned}$$
  where the first term is the unitary evolution with Hamiltonian $H_s$, the second term is the nonunitary contribution, $L_{i, \alpha }$ is the Lindblad operator acting on the *i*th qubit. In order to simplify the calculation, the evolution of $\hat{\rho }$ can be considered first before calculating the result of the whole evolved state $\rho$ (see “Methods” for detail).
*Step 3*: Alice makes a von Neumann type measurement on her own qubits *a*, 1, 2 by using the proper states $\{|\psi _j\rangle , j=1, 2, 3, 4\}$. After this measurement, the total state $\rho (t)$ changes to one of the following four states with a certain probability.
  6 $$\begin{aligned} \begin{aligned} \rho _j(t)=\frac{M_j \rho (t)M_j^\dagger }{\textrm{Tr}[M_j^\dagger M_j\rho (t)]}, \end{aligned} \end{aligned}$$
  where Tr is the trace operation and $M_j=|\psi _j\rangle \langle \psi _j|.$ In this step, we consider the measurement is perfect, while the particles still suffer from the noise during the measurement.
*Step 4*: Alice tells the measurement outcome to Bob through classical communication. Now, Bob knows his own state is given by
  7 $$\begin{aligned} \begin{aligned} \rho _{Bj}(t)=\textrm{Tr}_{a12}[\rho _j(t)]=\frac{\textrm{Tr}_{a12}[M_j\rho (t)M_j^\dagger ]}{\textrm{Tr}[M_j^\dagger M_j\rho (t)]}, \end{aligned} \end{aligned}$$
  where $\textrm{Tr}_{a12}$ is the partial trace over particles (*a*, 1, 2).
*Step 5*: Bob implements a unitary operator $U_j$ on his particle 3. Hence, the final state in Bob is
  8 $$\begin{aligned} \begin{aligned} \tilde{\rho }_{Bj}(t)=U_j\rho _{Bj}(t)U_j^\dagger =\frac{U_j\textrm{Tr}_{a12}[ M_j\rho (t)M_j^\dagger ]U_j^\dagger }{\textrm{Tr}[M_j^\dagger M_j\rho (t)]}. \end{aligned} \end{aligned}$$
  To describe how much information has been lost in this transmission, we compute the teleportation fidelity, which indicates the degree of overlap between the input and output states. The fidelity of quantum teleportation is
  9 $$\begin{aligned} F=\sum _{j=1}^4P_jF_j, \end{aligned}$$
  where $$P_j={\textrm{Tr}[M_j^\dagger M_j\rho (t)]}$$ and $$F_j=\langle \varphi _a|\tilde{\rho }_{Bj}(t)|\varphi _a\rangle .$$

  

In addition, one way to protect entanglement is based on WM. It was found that the process of performing WM and its reversal on individual qubit is a very useful technique for preserving and enhancing correlations under noisy conditions^33–36^. Furthermore, WM and RM have also been experimentally demonstrated^37,38^. So, we further investigate the effect of WM and RM on protocol effectiveness during teleportation. The modified QT under noises with the help of WM and RM is described as follows.

We start with a scenario where Dave prepares a three-qubit pure entangled state $|\phi \rangle _{123}$. The form of the whole initial system is Eq. (4). To reduce the effect of decoherence, suppose that Dave performs WM on each qubit before sending the particle through noisy environments. Here, the weak measurement operator is

10 $$\begin{aligned} \begin{aligned} W_{w}=\left( \begin{array}{cc} 1 &{} 0\\ 0&{} \sqrt{1-w} \\ \end{array} \right) , \end{aligned} \end{aligned}$$

where *w* is the strength of WM operations and $0\le w<1$. According to the operators $W_{w}$ of WM, the initial quantum system is rewritten as

11 $$\begin{aligned} \begin{aligned} \rho ^{wk} & = \frac{W\rho W^\dagger }{\textrm{Tr}[W^\dagger W\rho ]}. \end{aligned} \end{aligned}$$

where $W=W_1W_2W_3$ and $W_1=I^{(a)}\otimes W_{w_1}^{(1)}\otimes I^{(2)}\otimes I^{(3)}, W_2=I^{(a)}\otimes I^{(1)}\otimes W_{w_2}^{(2)}\otimes I^{(3)}, W_3=I^{(a)}\otimes I^{(1)}\otimes I^{(2)} \otimes W_{w_3}^{(3)},$ $w_1, w_2$ and $w_3$ are the strengths of WM on qubit 1, 2, 3, respectively. $\textrm{Tr}[W^\dagger W\rho ]$ is the success probability of the weak measurement.

Then, Dave sends particles through noise environments. The evolved state $\rho ^{wk}(t)$ can be given by solving the master equation Eq. (5) according to the conditions that quantum noise and initial quantum state.

After receiving the qubits, Alice and Bob perform RM on qubit (1, 2) and qubit 3, respectively. The quantum reversal measurement operator is^37^

12 $$\begin{aligned} \begin{aligned} R_{r}=\left( \begin{array}{cc} \sqrt{1-r} &{} 0\\ 0&{} 1\\ \end{array} \right) , \end{aligned} \end{aligned}$$

where *r* represents the strength of RM operations $0\le r<1$. The quantum system becomes

13 $$\begin{aligned} \begin{aligned} \rho ^{rev}(t) & = \frac{R \rho ^{wk}(t) R^\dagger }{{\textrm{Tr}}[R^\dagger R\rho^{wk}(t) ]}, \end{aligned} \end{aligned}$$

where $R=R_1R_2R_3$ and $R_1=I^{(a)}\otimes R_{r_1}^{(1)}\otimes I^{(2)}\otimes I^{(3)}, R_2=I^{(a)}\otimes I^{(1)}\otimes R_{r_2}^{(2)}\otimes I^{(3)}, R_3=I^{(a)}\otimes I^{(1)}\otimes I^{(2)} \otimes R_{r_3}^{(3)},$ $r_1, r_2$ and $r_3$ are the strengths of RM respectively performs on qubit 1, 2 and 3. The probability of performing a successful RM operation is ${\textrm{Tr}}[R^\dagger R\rho^{wk}(t)].$

Finally, as in step 3 to step 5, Alice and Bob complete teleportation by using the entangled state $\rho ^{rev}(t)$. The fidelity of the quantum teleportation using three-qubit state with the help of WK and RM is

14 $$\begin{aligned} \begin{aligned} F=\sum _{j=1}^4 U_j\textrm{Tr}_{a12}[M_j\rho ^{rev}(t)M_j^\dagger ]U_j^\dagger . \end{aligned} \end{aligned}$$

### Quantum teleportation with GHZ state and $W_1$ state

In general, three-qubit entangled states are classified into two inequivalent classes, namely GHZ class and *W* class. There is an instance in three-qubit entangled state where the maximally entangled GHZ state can be used for deterministic teleportation, while the standard *W* state can only be used for probabilistic teleportation. Interestingly, Agrawal et al.^7^ showed that there exists a special class of *W* states that can be used for perfect teleportation, called $W_n$. Therefore, we consider GHZ and $W_n$ states in the following.

First, the situation of GHZ state is considered. Suppose Dave prepares a GHZ state $|\phi _g\rangle _{123}$, which is

15 $$\begin{aligned} |\phi _g\rangle _{123}=\frac{1}{\sqrt{2}}(|000\rangle +|111\rangle )_{123}. \end{aligned}$$

The density matrix of $|\phi _g\rangle _{123}$ is denoted by $\hat{\rho }_g(0)=|\phi _g\rangle _{123}\langle \phi _g|.$

Here, we consider the evolution of the state $\hat{\rho }_g(0)$ under amplitude damping noise, which describes the energy dissipation. Equation (46) provides a specific description. By substituting coefficients of $|\phi _g\rangle _{123}$ into Eq. (52), that is, $\lambda _0=\lambda _1=\frac{1}{\sqrt{2}}$, one can obtain the evolved state of $|\phi _g\rangle _{123}$, which is the mixed state shared by Alice and Bob.

After Dave sends particles through amplitude damping noise, the whole quantum state has the following form,

16 $$\begin{aligned} \begin{aligned} \rho _g(t) & =\rho _{in}\otimes \hat{\rho }_g(t)\\ & =\frac{1}{2}\left[ \left( \alpha |0000\rangle +\alpha e^{\frac{-k_1t}{2}}e^{\frac{-k_2t}{2}}e^{\frac{-k_3t}{2}}|0111\rangle +\beta |1000\rangle +\beta e^{\frac{-k_1t}{2}}e^{\frac{-k_2t}{2}}e^{\frac{-k_3t}{2}}|1111\rangle \right) \right. \\&\left( \alpha \langle 0000|+\alpha e^{\frac{-k_1t}{2}}e^{\frac{-k_2t}{2}} e^{\frac{-k_3t}{2}}\langle 0111|+\beta \langle 1000|+\beta e^{\frac{-k_1t}{2}} e^{\frac{-k_2t}{2}}e^{\frac{-k_3t}{2}}\langle 1111|\right) \\ & \quad +e^{-k_1t}e^{-k_2t}\bar{e}^{-k_3t}\left( \alpha |0110\rangle +\beta |1110\rangle \right) \left( \alpha \langle 0110|+\beta \langle 1110|\right) \\ & \quad +e^{-k_1t}\bar{e}^{-k_2t}e^{-k_3t} \left( \alpha |0101\rangle +\beta |1101\rangle \right) \left( \alpha \langle 0101|+\beta \langle 1101|\right) \\ & \quad +e^{-k_1t}\bar{e}^{-k_2t}\bar{e}^{-k_3t} \left( \alpha |0100\rangle +\beta |1100\rangle \right) \left( \alpha \langle 0100|+\beta \langle 1100|\right) \\ & \quad +\bar{e}^{-k_1t}e^{-k_2t}e^{-k_3t} \left( \alpha |0011\rangle +\beta |1011\rangle \right) \left( \alpha \langle 0011|+\beta \langle 1011|\right) \\ & \quad +\bar{e}^{-k_1t}e^{-k_2t}\bar{e}^{-k_3t} \left( \alpha |0010\rangle +\beta |1010\rangle \right) \left( \alpha \langle 0010|+\beta \langle 1010|\right) \\ & \quad +\bar{e}^{-k_1t}\bar{e}^{-k_2t}e^{-k_3t} \left( \alpha |0001\rangle +\beta |1001\rangle \right) \left( \alpha \langle 0001|+\beta \langle 1001|\right) \\ & \quad \left. +\bar{e}^{-k_1t}\bar{e}^{-k_2t}\bar{e}^{-k_3t} \left( \alpha |0000\rangle +\beta |1000\rangle \right) \left( \alpha \langle 0000|+\beta \langle 1000|\right) \right] , \end{aligned} \end{aligned}$$

where $k_1, k_2$ and $k_3$ are the amplitude damping parameters associated with particles 1, 2 and 3, respectively. And the detailed calculation procedure of $\hat{\rho }_g(0)$ can be found in the “Methods”-“GHZ state”.

Now, Alice makes a von Neumann measurement on particles (*a*, 1, 2) with a set of orthogonal states given by

17 $$\begin{aligned} \begin{aligned} |\psi _1\rangle =\frac{1}{\sqrt{2}}(|000\rangle +|111\rangle ), |\psi _2\rangle =\frac{1}{\sqrt{2}}(|000\rangle -|111\rangle ), |\psi _3\rangle =\frac{1}{\sqrt{2}}(|100\rangle +|011\rangle ), |\psi _4\rangle =\frac{1}{\sqrt{2}}(|100\rangle -|011\rangle ). \end{aligned} \end{aligned}$$

Subsequently, Alice sends the result of her measurement to Bob by using classical communication with two bits. According to the measurement result, Bob applies a proper unitary operator to recover the state of his particle 3 to that of state $|\varphi \rangle _a$. Hence, one can obtain the output quantum state

18 $$\begin{aligned} \begin{aligned} \rho _g^{out}(t)& = \sum _{j=1}^4\textrm{Tr}[M_j^\dagger M_j\rho (t)]\frac{U_j\textrm{Tr}_{a12}[M_j\rho (t)M_j^\dagger ]U_j^\dagger }{\textrm{Tr}[M_j^\dagger M_j\rho (t)]}\\ & = \frac{1}{2}\left[ \left( \alpha |0\rangle +\beta e^{\frac{-k_1t}{2}}e^{\frac{-k_2t}{2}}e^{\frac{-k_3t}{2}}|1\rangle \right) \left( \alpha \langle 0|+\beta e^{\frac{-k_1t}{2}}e^{\frac{-k_2t}{2}}e^{\frac{-k_3t}{2}}\langle 1|\right) \right. \\ & \quad +\left( \alpha e^{\frac{-k_1t}{2}}e^{\frac{-k_2t}{2}}e^{\frac{-k_3t}{2}}|0\rangle +\beta |1\rangle \right) \left( \alpha e^{\frac{-k_1t}{2}}e^{\frac{-k_2t}{2}}e^{\frac{-k_3t}{2}}\langle 0|+\beta \langle 1|\right) \\ & \quad +\beta ^2e^{-k_1t}e^{-k_2t}\bar{e}^{-k_3t}|0\rangle \langle 0|+\alpha ^2\bar{e}^{-k_1t} \bar{e}^{-k_2t}e^{-k_3t}|1\rangle \langle 1|\\ & \quad +\alpha ^2\bar{e}^{-k_1t}\bar{e}^{-k_2t}\bar{e}^{-k_3t}|0\rangle \langle 0| +\alpha ^2e^{-k_1t}e^{-k_2t}\bar{e}^{-k_3t}|1\rangle \langle 1|\\& \quad \left. +\beta ^2\bar{e}^{-k_1t}\bar{e}^{-k_2t}e^{-k_3t}|0\rangle \langle 0| +\beta ^2\bar{e}^{-k_1t}\bar{e}^{-k_2t}\bar{e}^{-k_3t}|1\rangle \langle 1|\right] . \end{aligned} \end{aligned}$$

where $$M_j=|\psi _j\rangle \langle \psi _j|$$, $$U_j=\sigma _j, j=1, 2, 3, 4$$, and $$\sigma _1=\left( \begin{array}{cc} 1 &{} 0\\ 0&{} 1 \\ \end{array} \right) , \sigma _2=\left( \begin{array}{cc} 1 &{} 0\\ 0&{} -1 \\ \end{array} \right) , \sigma _3=\left( \begin{array}{cc} 0 &{} 1\\ 1&{} 0\\ \end{array} \right) , \sigma _4=\left( \begin{array}{cc} 0 &{} -1\\ 1&{} 0 \\ \end{array} \right)$$.

Based on Eq. (9), the fidelity of the quantum teleportation using GHZ state as a quantum channel is

19 $$\begin{aligned} \begin{aligned} F_g& = \frac{1}{2}\left[ \left( \alpha ^4+\beta ^4\right) \left( 1+e^{\frac{-k_1t}{2}}e^{\frac{-k_2t}{2}} e^{\frac{-k_3t}{2}}+\bar{e}^{-k_1t}\bar{e}^{-k_2t}\bar{e}^{-k_3t}\right) \right] +\alpha ^2\beta ^2\left( 2e^{\frac{-k_1t}{2}}e^{\frac{-k_2t}{2}}e^{\frac{-k_3t}{2}} +e^{-k_1t}e^{-k_2t}\bar{e}^{-k_3t}+\bar{e}^{-k_1t}\bar{e}^{-k_2t}\bar{e}^{-k_3t}\right) . \end{aligned} \end{aligned}$$

We next consider another class of three-qubit entangled state named *W* state. The standard *W* state can only be used for probabilistic teleportation. In Re^7^, Agrawal and Pati have shown that there exists a special class of *W* state can be used for perfect teleportation. We consider an example of this class non-standard *W* state

20 $$\begin{aligned} |W_1\rangle _{123}=\frac{1}{2}\left( |100\rangle +|010\rangle +\sqrt{2}|001\rangle \right) _{123}. \end{aligned}$$

Similarly, we assume that Dave prepares an entangled state $|W_1\rangle _{123}$, then transmits particles (1, 2) and 3 to Alice and Bob through the amplitude damping noise environment, respectively. The initial state is

21 $$\begin{aligned} \begin{aligned} \rho _{W_1}(0)& = |\varphi \rangle _a\langle \varphi |_a\otimes |W_1\rangle _{123}\langle W_1|_{123}. \end{aligned} \end{aligned}$$

According to Eq. (59), let $x=\frac{1}{\sqrt{2}}, y=\frac{1}{2}, z=\frac{1}{2}$, one can obtain the expression of the total evolution quantum state, which is

22 $$\begin{aligned} \begin{aligned} \rho _{W_1}(t)& = \frac{1}{4}\left( \alpha e^{\frac{-k_1t}{2}}|0100\rangle +\alpha e^{\frac{-k_2t}{2}}|0010\rangle +\sqrt{2}\alpha e^{\frac{-k_3t}{2}}|0001\rangle +\beta e^{\frac{-k_1t}{2}}|1100\rangle \right. \\ & \quad \left. +\beta e^{\frac{-k_2t}{2}}|1010\rangle +\sqrt{2}\beta e^{\frac{-k_3t}{2}}|1001\rangle \right) \left( \alpha e^{\frac{-k_1t}{2}}\langle 0100| +\alpha e^{\frac{-k_2t}{2}}\langle 0010|\right. \\ & \quad \left. +\sqrt{2}\alpha e^{\frac{-k_3t}{2}}\langle 0001|+\beta e^{\frac{-k_1t}{2}}\langle 1100|+\beta e^{\frac{-k_2t}{2}}\langle 1010| +\sqrt{2}\beta e^{\frac{-k_3t}{2}}\langle 1001|\right) \\ & \quad +\left[ 2\bar{e}^{-k_3t}+\bar{e}^{-k_2t}+\bar{e}^{-k_1t}\right] \left( \alpha |0000\rangle +\beta |1000\rangle \right) \left( \alpha \langle 0000|+\beta \langle 1000|\right) . \end{aligned} \end{aligned}$$

Then, Alice makes a von Neumann measurement on qubit (*a*, 1, 2) using the states $\{|\zeta _j\rangle , j=1, 2, 3, 4\}$, which is

23 $$\begin{aligned} \begin{aligned} |\zeta _1\rangle =\frac{1}{2}\left( |010\rangle +|001\rangle +\sqrt{2}|100\rangle \right) , |\zeta _2\rangle =\frac{1}{2}\left( |010\rangle +|001\rangle -\sqrt{2}|100\rangle \right) ,\\ |\zeta _3\rangle =\frac{1}{2}\left( |110\rangle +|101\rangle +\sqrt{2}|000\rangle \right) , |\zeta _4\rangle =\frac{1}{2}\left( |110\rangle +|101\rangle -\sqrt{2}|000\rangle \right) . \end{aligned} \end{aligned}$$

After measurement, Alice sends measurement result to Bob by classical communication. Bob performs a unitary operator on his own qubit 3. The final state of Bob is

24 $$\begin{aligned} \begin{aligned} \rho _{W_1}^{out}(t)& = \sum _{j=1}^4V_j\textrm{Tr}_{a12}[N_j^\dagger N_j \rho _{W_1}(t)N_j^\dagger ]V_j^\dagger \\ & = \frac{1}{8}\left\{ \left[ \left( e^{\frac{-k_1t}{2}}+e^{\frac{-k_2t}{2}}\right) \alpha |0\rangle +2e^{\frac{-k_3t}{2}}\beta |1\rangle \right] \left[ \left( e^{\frac{-k_1t}{2}} +e^{\frac{-k_2t}{2}}\right) \alpha \langle 0| +2e^{\frac{-k_3t}{2}}\beta \langle 1|\right] \right. \\ & \quad +\left[ 2e^{\frac{-k_3t}{2}}\alpha |0\rangle +\left( e^{\frac{-k_2t}{2}} +e^{\frac{-k_1t}{2}}\right) \beta |1\rangle \right] \left[ 2e^{\frac{-k_3t}{2}}\alpha \langle 0| +\left( e^{\frac{-k_2t}{2}}+e^{\frac{-k_1t}{2}}\right) \beta \langle 1|\right] \\ & \quad \left. +2\beta ^2\left( 2\bar{e}^{-k_3t}+\bar{e}^{-k_2t}+\bar{e}^{-k_1t}\right) |0\rangle \langle 0| +2\alpha ^2\left( 2\bar{e}^{-k_3t}+\bar{e}^{-k_2t}+\bar{e}^{-k_1t}\right) |1\rangle \langle 1|\right\} , \end{aligned} \end{aligned}$$

where $$V_j=U_j$$ and $$N_j=|\zeta _j\rangle \langle \zeta _j|.$$

Using fidelity to measure the amount of information loss in the quantum teleportation process, which can be written as

25 $$\begin{aligned} \begin{aligned} F_{W_1}& = \frac{1}{8}\left\{ \left[ \left( e^{\frac{-k_1t}{2}}+e^{\frac{-k_2t}{2}}\right) \alpha ^2+2e^{\frac{-k_3t}{2}}\beta ^2\right] ^2+\left[ 2e^{\frac{-k_3t}{2}} \alpha ^2+\left( e^{\frac{-k_1t}{2}}+e^{\frac{-k_2t}{2}}\right) \beta ^2\right] ^2 +4\alpha ^2\beta ^2\left( 2\bar{e}^{-k_3t}+\bar{e}^{-k_2t}+\bar{e}^{-k_1t}\right) \right\} . \end{aligned} \end{aligned}$$

**Figure 1:**
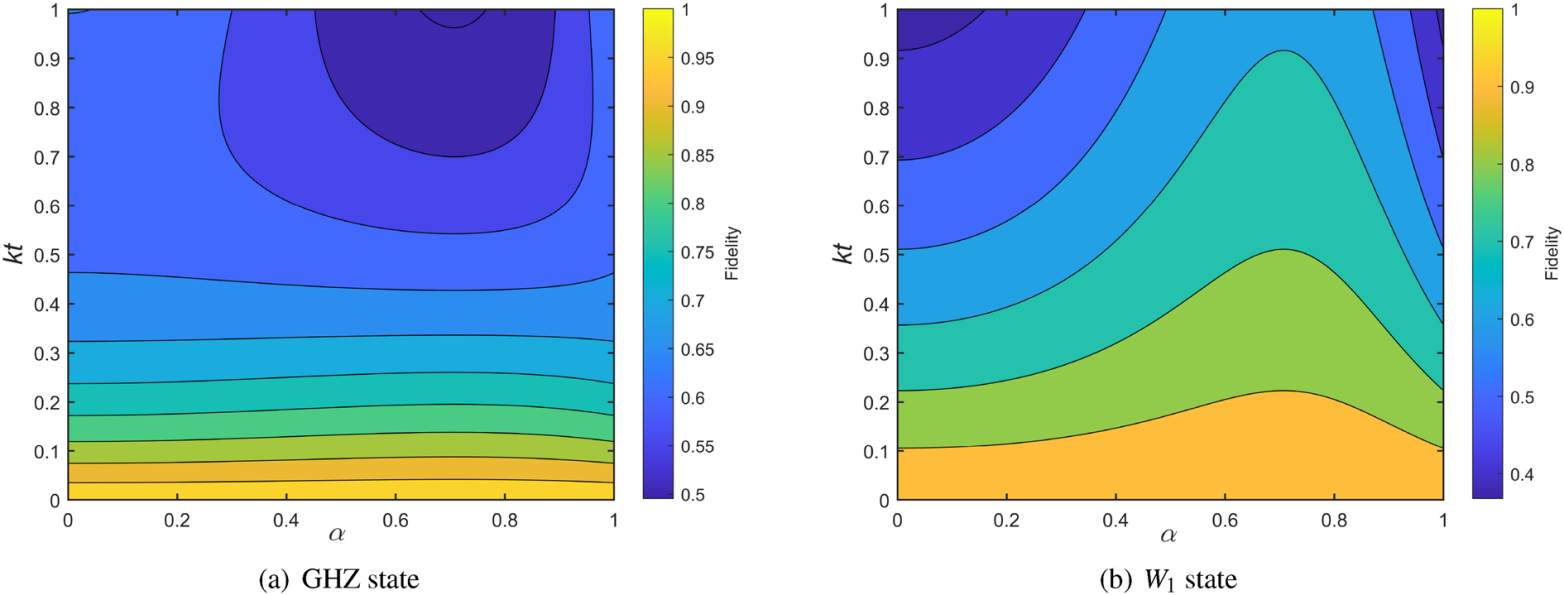
The fidelities of quantum teleportation using GHZ and $W_1$ state under amplitude damping noise is described by a master equation in the Lindblad form.

We consider a situation where the particles to be sent are affected by the same noise parameters, namely, $k_1=k_2=k_3=k$. In order to describe the relationship between the initial state and the fidelity more vividly, we carried out a simulation and plotted the Fig. 1. In reality, the measurement time needs to be considered, but we assume that the measurement time is extremely small in the numerical simulation. Obviously, Dave sends qubits through the perfect channel, that is $kt=0$, then the fidelities of GHZ and $W_1$ state are both 1. It also depicts that whether GHZ state or $W_1$ state is used as a quantum channel in the process of quantum teleportation, the fidelity of the final quantum state obtained by the receiver Bob will decrease with the increase of decoherence time. For GHZ state, as shown in plot (a), the state of the input state has little effect on the fidelity of the final output state when $kt\le 0.5$. If *kt* continues to increase, the state of the transmission state will affect the fidelity of the transmission. In this case where the smaller the value of $\alpha$, the higher the fidelity of teleportation. Plot (b) describes the effects of decoherence time and input state on the value of teleportation fidelity of $W_1$ state. The value of teleportation fidelity of $W_1$ is always greater than 0.9, if $kt\le 0.1.$ Unlike the former, the fidelity of $W_1$ is affected not only by decoherence time, but also by the input state when $kt\le 0.5$. Clearly, the fidelity will be affected by the unknown state as the value of *kt* increases. When $0.6<\alpha <0.8$, the efficiency of protocol performs better. At this time, even *kt* reaches 0.9, the fidelity $F_{W_1}\ge 0.6$.

**Figure 2:**
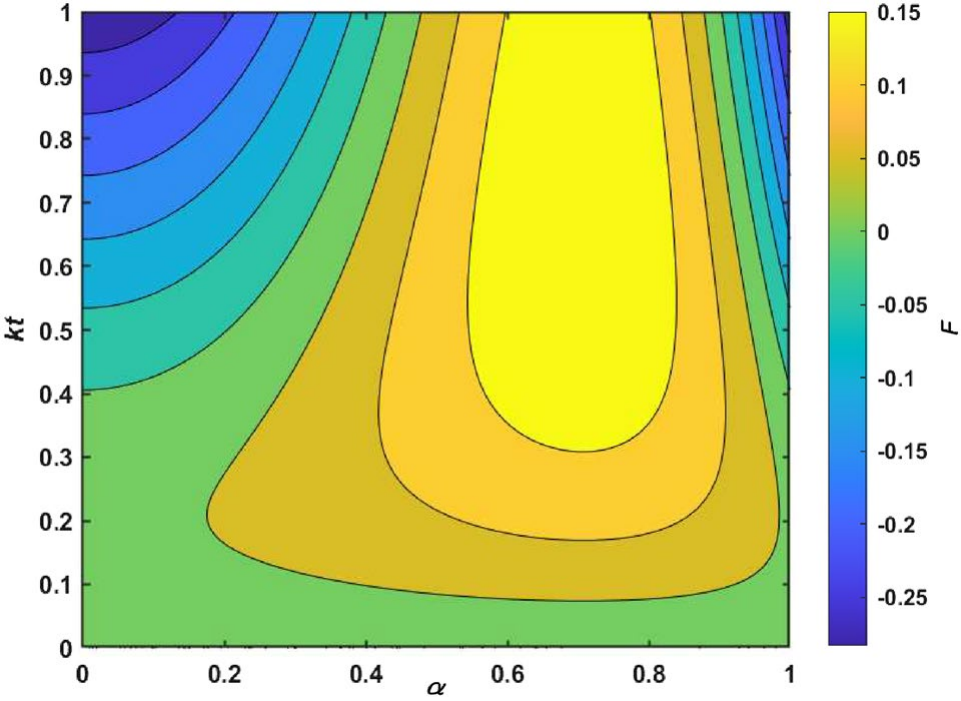
The comparison of fidelities of quantum teleportation between GHZ state and $W_1$ state. Considering the noise parameters of GHZ and $W_1$ both are $k_1=k_2=k_3=k$.

Furthermore, we make a comparison between GHZ state and $W_1$ state in the same condition. The fidelity of GHZ ($F_g$) subtracted from $W_1$ ($F_{W_1}$) is denoted as *F*. As shown in Fig. 2, we found that the fidelity of $W_1$ is higher than GHZ, in most cases. The result obtained here confirms that the ability of $W_1$ to resist amplitude damping noise during the communication process is stronger than GHZ state.

It is worth to discuss the feasibility of the protocol in physical experiments. QT has been demonstrated in different systems such as photonic qubit, continuous variables, nuclear magnetic resonance, atomic ensembles, trapped ions and solid state systems^39^. Combined with the development trend of quantum communication, transmission distance is one of the key points. Hence, photon is the most suitable carrier of information for the long distance QT. In the numerical simulations, the value of *t* depends on the value of *k*. Theoretically, the particles can reach their destinations in a finite time by choosing the appropriate *k*. In the aspect of experiment, since there are lots of works have been experimentally demonstrated the feasibility of the QT protocol employing photonic qubits^5,40–42^, it is possible to distribute the entangled particles to the communicating parties even if the decoherence time is very short.

### Quantum teleportation with WM and RM

Let us now consider the efficiency of QT protocol with the help of WM and RM operations under noises, by using GHZ state. We assume that Dave prepares a three qubit entangled state $|\phi _g\rangle$. Then, Dave performs WM on qubits 1, 2, 3 before sending them to Alice and Bob. According to the operators $W_{wk}$ of WM, the initial entangled quantum state is became

26 $$\begin{aligned} \begin{aligned} \rho ^{wk}_g(0)& = W\rho _g(0)W\\ & = \frac{1}{2}\left( |000\rangle +\bar{w}_1^{\frac{1}{2}}\bar{w}_2^{\frac{1}{2}}\bar{w}_3^{\frac{1}{2}} |111\rangle \right) \left( \langle 000|+\bar{w}_1^{\frac{1}{2}}\bar{w}_2^{\frac{1}{2}}\bar{w}_3^{\frac{1}{2}} \langle 111|\right) , \end{aligned} \end{aligned}$$

where $W=W_1W_2W_3$ and $W_1=W_{w_1k}^{(1)}\otimes I^{(2)}\otimes I^{(3)}, W_2=I^{(1)}\otimes W_{w_2k}^{(2)}\otimes I^{(3)}, W_3=I^{(1)}\otimes I^{(2)} \otimes W_{w_3k}^{(3)},$ $\bar{w}_1=1-w_1, \bar{w}_2=1-w_2, \bar{w}_3=1-w_3, w_1, w_2$ and $w_3$ are the strengths of WM on qubits 1, 2, 3, respectively.

Dave sends particles through amplitude damping noise channel. The evolved state $\hat{\rho }_g^{wk}(t)$ can be given refer to Eq. (52) with the parameter in Eq. (26). It is

27 $$\begin{aligned} \begin{aligned} \hat{\rho }_g^{wk}(t)& = \frac{1}{2}\left[ \left( |000\rangle +\bar{w}_1^{\frac{1}{2}}\bar{w}_2^{\frac{1}{2}} \bar{w}_3^{\frac{1}{2}}e^{\frac{-k_1t}{2}}e^{\frac{-k_2t}{2}}e^{\frac{-k_3t}{2}}|111\rangle \right) \left( \langle 000|+\bar{w}_1^{\frac{1}{2}}\bar{w}_2^{\frac{1}{2}}\bar{w}_3^{\frac{1}{2}} e^{\frac{-k_1t}{2}}e^{\frac{-k_2t}{2}}e^{\frac{-k_3t}{2}}\langle 111|\right) \right. \\ & \quad +\bar{w}_1\bar{w}_2\bar{w}_3e^{-k_1t}e^{-k_2t}\bar{e}^{-k_3t}|110\rangle \langle 110| +\bar{w}_1\bar{w}_2\bar{w}_3e^{-k_1t}\bar{e}^{-k_2t}e^{-k_3t}|101\rangle \langle 101|\\ & \quad +\bar{w}_1\bar{w}_2\bar{w}_3e^{-k_1t}\bar{e}^{-k_2t}\bar{e}^{-k_3t}|100\rangle \langle 100| +\bar{w}_1\bar{w}_2\bar{w}_3\bar{e}^{-k_1t}e^{-k_2t}e^{-k_3t}|011\rangle \langle 011|\\ & \quad +\bar{w}_1\bar{w}_2\bar{w}_3\bar{e}^{-k_1t}e^{-k_2t}\bar{e}^{-k_3t}|010\rangle \langle 010| +\bar{w}_1\bar{w}_2\bar{w}_3\bar{e}^{-k_1t}\bar{e}^{-k_2t}e^{-k_3t}|001\rangle \langle 001|\\ & \quad \left. +\bar{w}_1\bar{w}_2\bar{w}_3\bar{e}^{-k_1t}\bar{e}^{-k_2t}\bar{e}^{-k_3t}|000\rangle \langle 000|\right] . \end{aligned} \end{aligned}$$

Subsequently, Alice and Bob perform RM on qubit (1, 2) and qubit 3, respectively. By Eq. (13), the three-qubit entangled state is changed to

28 $$\begin{aligned} \begin{aligned} \hat{\rho }_g^{rev}(t)& = \frac{1}{2}\left[ \left( \bar{r}_1^{\frac{1}{2}}\bar{r}_2^{\frac{1}{2}} \bar{r}_3^{\frac{1}{2}}|000\rangle +\bar{w}_1^{\frac{1}{2}}\bar{w}_2^{\frac{1}{2}} \bar{w}_3^{\frac{1}{2}}e^{\frac{-k_1t}{2}}e^{\frac{-k_2t}{2}}e^{\frac{-k_3t}{2}}|111\rangle \right) \left( \bar{r}_1^{\frac{1}{2}}\bar{r}_2^{\frac{1}{2}}\bar{r}_3^{\frac{1}{2}}\langle 000| +\bar{w}_1^{\frac{1}{2}}\bar{w}_2^{\frac{1}{2}}\bar{w}_3^{\frac{1}{2}} e^{\frac{-k_1t}{2}}e^{\frac{-k_2t}{2}}e^{\frac{-k_3t}{2}}\langle 111|\right) \right. \\ & \quad +\bar{w}_1\bar{w}_2\bar{w}_3e^{-k_1t}e^{-k_2t}\bar{e}^{-k_3t}\bar{r}_3|110\rangle \langle 110| +\bar{w}_1\bar{w}_2\bar{w}_3e^{-k_1t}\bar{e}^{-k_2t}e^{-k_3t}\bar{r}_2|101\rangle \langle 101|\\ & \quad +\bar{w}_1\bar{w}_2\bar{w}_3e^{-k_1t}\bar{e}^{-k_2t}\bar{e}^{-k_3t}\bar{r}_2\bar{r}_3|100\rangle \langle 100|+\bar{w}_1\bar{w}_2\bar{w}_3\bar{e}^{-k_1t}e^{-k_2t}e^{-k_3t}\bar{r}_1|011\rangle \langle 011|\\ & \quad +\bar{w}_1\bar{w}_2\bar{w}_3\bar{e}^{-k_1t}e^{-k_2t}\bar{e}^{-k_3t}\bar{r}_1\bar{r}_3|010\rangle \langle 010|+\bar{w}_1\bar{w}_2\bar{w}_3\bar{e}^{-k_1t}\bar{e}^{-k_2t}e^{-k_3t}\bar{r}_1 \bar{r}_2|001\rangle \langle 001|\\ & \quad \left. +\bar{w}_1\bar{w}_2\bar{w}_3\bar{e}^{-k_1t}\bar{e}^{-k_2t}\bar{e}^{-k_3t}\bar{r}_1\bar{r}_2 \bar{r}_3|000\rangle \langle 000|\right] , \end{aligned} \end{aligned}$$

where $\bar{r}_1=1-r_1, \bar{r}_2=1-r_2, \bar{r}_3=1-r_3$, $r_1, r_2$ and $r_3$ are the strengths of RM respectively performs on qubit 1, 2 and 3.

Now, Alice and Bob share a mixed state $\hat{\rho }_g^{rev}(t)$, which is used to transmit the unknown single-qubit state. Obviously, the total quantum system is

29 $$\begin{aligned} \begin{aligned} \rho _g^{wk}(t)& = \rho _{in}\otimes \hat{\rho }_g^{rev}(t)\\ & = \left( \alpha \lambda _0|0000\rangle +\alpha \lambda _1\bar{w}_1^{\frac{1}{2}}\bar{w}_2^{\frac{1}{2}} \bar{w}_3^{\frac{1}{2}} e^{\frac{-k_1t}{2}}e^{\frac{-k_2t}{2}}e^{\frac{-k_3t}{2}}|0111\rangle +\beta \lambda _0|1000\rangle +\beta \lambda _1\bar{w}_1^{\frac{1}{2}}\bar{w}_2^{\frac{1}{2}}\bar{w}_3^{\frac{1}{2}}\right. \\ & \quad \left. e^{\frac{-k_1t}{2}}e^{\frac{-k_2t}{2}}e^{\frac{-k_3t}{2}}|1111\rangle \right) \left( \alpha \lambda _0\langle 0000|+\alpha \lambda _1\bar{w}_1^{\frac{1}{2}}\bar{w}_2^{\frac{1}{2}} \bar{w}_3^{\frac{1}{2}} e^{\frac{-k_1t}{2}}e^{\frac{-k_2t}{2}}e^{\frac{-k_3t}{2}}\langle 0111|\right. \\ & \quad \left. +\beta \lambda _0\langle 1000|+\beta \lambda _1\bar{w}_1^{\frac{1}{2}}\bar{w}_2^{\frac{1}{2}} \bar{w}_3^{\frac{1}{2}}e^{\frac{-k_1t}{2}}e^{\frac{-k_2t}{2}} e^{\frac{-k_3t}{2}}\langle 1111|\right) \\ & \quad +\lambda _1^2\bar{w}_1\bar{w}_2\bar{w}_3e^{-k_1t}e^{-k_2t}\bar{e}^{-k_3t} \left( \alpha |0110\rangle +\beta |1110\rangle \right) \left( \alpha \langle 0110|+\beta \langle 1110|\right) \\ & \quad +\lambda _1^2\bar{w}_1\bar{w}_2\bar{w}_3e^{-k_1t}\bar{e}^{-k_2t}e^{-k_3t} \left( \alpha |0101\rangle +\beta |1101\rangle \right) \left( \alpha \langle 0101|+\beta \langle 1101|\right) \\ & \quad +\lambda _1^2\bar{w}_1\bar{w}_2\bar{w}_3e^{-k_1t}\bar{e}^{-k_2t}\bar{e}^{-k_3t}\left( \alpha |0100\rangle +\beta |1100\rangle \right) \left( \alpha \langle 0100|+\beta \langle 1100|\right) \\ & \quad +\lambda _1^2\bar{w}_1\bar{w}_2\bar{w}_3\bar{e}^{-k_1t}e^{-k_2t}e^{-k_3t} \left( \alpha |0011\rangle +\beta |1011\rangle \right) \left( \alpha \langle 0011|+\beta \langle 1011|\right) \\ & \quad +\lambda _1^2\bar{w}_1\bar{w}_2\bar{w}_3\bar{e}^{-k_1t}e^{-k_2t}\bar{e}^{-k_3t}\left( \alpha |0010\rangle +\beta |1010\rangle \right) \left( \alpha \langle 0010|+\beta \langle 1010|\right) \\ & \quad +\lambda _1^2\bar{w}_1\bar{w}_2\bar{w}_3\bar{e}^{-k_1t}\bar{e}^{-k_2t}e^{-k_3t} \left( \alpha |0001\rangle +\beta |1001\rangle \right) \left( \alpha \langle 0001|+\beta \langle 1001|\right) \\ & \quad +\lambda _1^2\bar{w}_1\bar{w}_2\bar{w}_3\bar{e}^{-k_1t}\bar{e}^{-k_2t}\bar{e}^{-k_3t}\left( \alpha |0000\rangle +\beta |1000\rangle \right) \left( \alpha \langle 0000|+\beta \langle 1000|\right) . \end{aligned} \end{aligned}$$

In the subsequent steps, Alice makes a von Neumann type measurement on her own qubits *a*, 1, 2 with the set of orthogonal states given by Eq. (17) and tells the measurement outcome to Bob through classical communication. According to the measurement result, Bob implements a unitary operator $U_j$ on his particle 3. So, the final quantum state is

30 $$\begin{aligned} \begin{aligned} \rho _g^{rev^{out}}(t)& = \sum _{j=1}^4U_j\textrm{Tr}_{a12}[M_j\rho _g^{rev}(t)M_j^\dagger ]U_j^\dagger \\ & = \frac{1}{2}\left[ \left( \alpha \bar{r}_1^{\frac{1}{2}}\bar{r}_2^{\frac{1}{2}}\bar{r}_3^{\frac{1}{2}}|0\rangle +\beta \bar{w}_1^{\frac{1}{2}}\bar{w}_2^{\frac{1}{2}}\bar{w}_3^{\frac{1}{2}}e^{\frac{-k_1t}{2}} e^{\frac{-k_2t}{2}}e^{\frac{-k_3t}{2}}|1\rangle \right) \left( \alpha \bar{r}_1^{\frac{1}{2}}\bar{r}_2^{\frac{1}{2}} \bar{r}_3^{\frac{1}{2}}\langle 0|\right. \right. \\ & \quad \left. +\beta \bar{w}_1^{\frac{1}{2}}\bar{w}_2^{\frac{1}{2}}\bar{w}_3^{\frac{1}{2}} e^{\frac{-k_1t}{2}}e^{\frac{-k_2t}{2}}e^{\frac{-k_3t}{2}}\langle 1|\right) +\beta ^2\bar{w}_1\bar{w}_2 \bar{w}_3e^{-k_1t}e^{-k_2t}\bar{e}^{-k_3t}\bar{r}_3|0\rangle \langle 0|\\ & \quad +\alpha ^2\bar{w}_1\bar{w}_2\bar{w}_3\bar{e}^{-k_1t}\bar{e}^{-k_2t}e^{-k_3t}\bar{r}_1\bar{r}_2|1\rangle \langle 1|+\alpha ^2\bar{w}_1\bar{w}_2\bar{w}_3\bar{e}^{-k_1t}\bar{e}^{-k_2t}\bar{e}^{-k_3t}\\&\bar{r}_1\bar{r}_2\bar{r}_3|0\rangle \langle 0|+\left(\alpha \bar{w}_1^{\frac{1}{2}}\bar{w}_2^{\frac{1}{2}} \bar{w}_3^{\frac{1}{2}}e^{\frac{-k_1t}{2}}e^{\frac{-k_2t}{2}}e^{\frac{-k_3t}{2}}|0\rangle +\beta \bar{r}_1^{\frac{1}{2}}\bar{r}_2^{\frac{1}{2}}\bar{r}_3^{\frac{1}{2}}|1\rangle \right) \left( \alpha \bar{w}_1^{\frac{1}{2}}\bar{w}_2^{\frac{1}{2}}\bar{w}_3^{\frac{1}{2}}\right. \\&\left. e^{\frac{-k_1t}{2}}e^{\frac{-k_2t}{2}}e^{\frac{-k_3t}{2}}\langle 0| +\beta \bar{r}_1^{\frac{1}{2}}\bar{r}_2^{\frac{1}{2}}\bar{r}_3^{\frac{1}{2}}\langle 1|\right) +\alpha ^2\bar{w}_1\bar{w}_2\bar{w}_3e^{-k_1t}e^{-k_2t}\bar{e}^{-k_3t}\bar{r}_3|1\rangle \langle 1|\\ & \quad +\beta ^2\bar{w}_1\bar{w}_2\bar{w}_3\bar{e}^{-k_1t}\bar{e}^{-k_2t}e^{-k_3t}\bar{r}_1\bar{r}_2|0 \rangle \langle 0|+\beta ^2\bar{w}_1\bar{w}_2\bar{w}_3\bar{e}^{-k_1t}\bar{e}^{-k_2t}\bar{e}^{-k_3t}\\&\left. \bar{r}_1\bar{r}_2\bar{r}_3|1\rangle \langle 1|\right] . \end{aligned} \end{aligned}$$

The fidelity of the quantum teleportation using GHZ state with the help of WK and RM is

31 $$\begin{aligned} \begin{aligned} F_g^{rev}& = \frac{1}{2}\left[ \left( \alpha ^2\bar{r}_1^{\frac{1}{2}}\bar{r}_2^{\frac{1}{2}} \bar{r}_3^{\frac{1}{2}}+\beta ^2\bar{w}_1^{\frac{1}{2}}\bar{w}_2^{\frac{1}{2}} \bar{w}_3^{\frac{1}{2}}e^{\frac{-k_1t}{2}}e^{\frac{-k_2t}{2}}e^{\frac{-k_3t}{2}}\right) ^2 +\left( \alpha ^2\bar{w}_1^{\frac{1}{2}}\bar{w}_2^{\frac{1}{2}}\bar{w}_3^{\frac{1}{2}} e^{\frac{-k_1t}{2}}e^{\frac{-k_2t}{2}}e^{\frac{-k_3t}{2}}+\beta ^2\bar{r}_1^{\frac{1}{2}} \bar{r}_2^{\frac{1}{2}}\bar{r}_3^{\frac{1}{2}}\right) ^2\right. \\ & \quad \left. +2\alpha ^2\beta ^2\bar{w}_1\bar{w}_2\bar{w}_3\left( e^{-k_1t}e^{-k_2t}\bar{e}^{-k_3t} \bar{r}_3+\bar{e}^{-k_1t}\bar{e}^{-k_2t}e^{-k_3t}\bar{r}_1\bar{r}_2\right) +(\alpha ^4+\beta ^4) \bar{w}_1\bar{w}_2\bar{w}_3\bar{e}^{-k_1t}\bar{e}^{-k_2t}e^{-k_3t}\bar{r}_1\bar{r}_2\bar{r}_3\right] . \end{aligned} \end{aligned}$$

For $W_1$ state, the fidelity of the QT under amplitude damping noise with the help of WM and RM can be calculated by the same method as GHZ state. It is

32 $$\begin{aligned} \begin{aligned} F_{W_1}^{rev}& = \frac{1}{8}\left\{ \left[ (\bar{w}_1^{\frac{1}{2}}e^{\frac{-k_1t}{2}} \bar{r}_2^{\frac{1}{2}}\bar{r}_3^{\frac{1}{2}}+\bar{w}_2^{\frac{1}{2}} e^{\frac{-k_2t}{2}}\bar{r}_1^{\frac{1}{2}}\bar{r}_3^{\frac{1}{2}})\alpha ^2 +2\bar{w}_3^{\frac{1}{2}}e^{\frac{-k_3t}{2}}\bar{r}_1^{\frac{1}{2}}\bar{r}_2^{\frac{1}{2}} \beta ^2\right] ^2+\left[ 2\bar{w}_3^{\frac{1}{2}}e^{\frac{-k_3t}{2}}\bar{r}_1^{\frac{1}{2}}\bar{r}_2^{\frac{1}{2}} \alpha ^2+(\bar{w}_1^{\frac{1}{2}}e^{\frac{-k_1t}{2}}\bar{r}_2^{\frac{1}{2}} \bar{r}_3^{\frac{1}{2}}+\bar{w}_2^{\frac{1}{2}}e^{\frac{-k_2t}{2}} \bar{r}_1^{\frac{1}{2}}\bar{r}_3^{\frac{1}{2}})\beta ^2\right] ^2\right. \\ & \quad \left. +4\bar{r}_1\bar{r}_2\bar{r}_3\alpha ^2\beta ^2\left( 2\bar{w}_3\bar{e}^{-k_3t} +\bar{w}_2\bar{e}^{-k_2t}+\bar{w}_1\bar{e}^{-k_1t}\right) \right\} . \end{aligned} \end{aligned}$$

**Figure 3:**
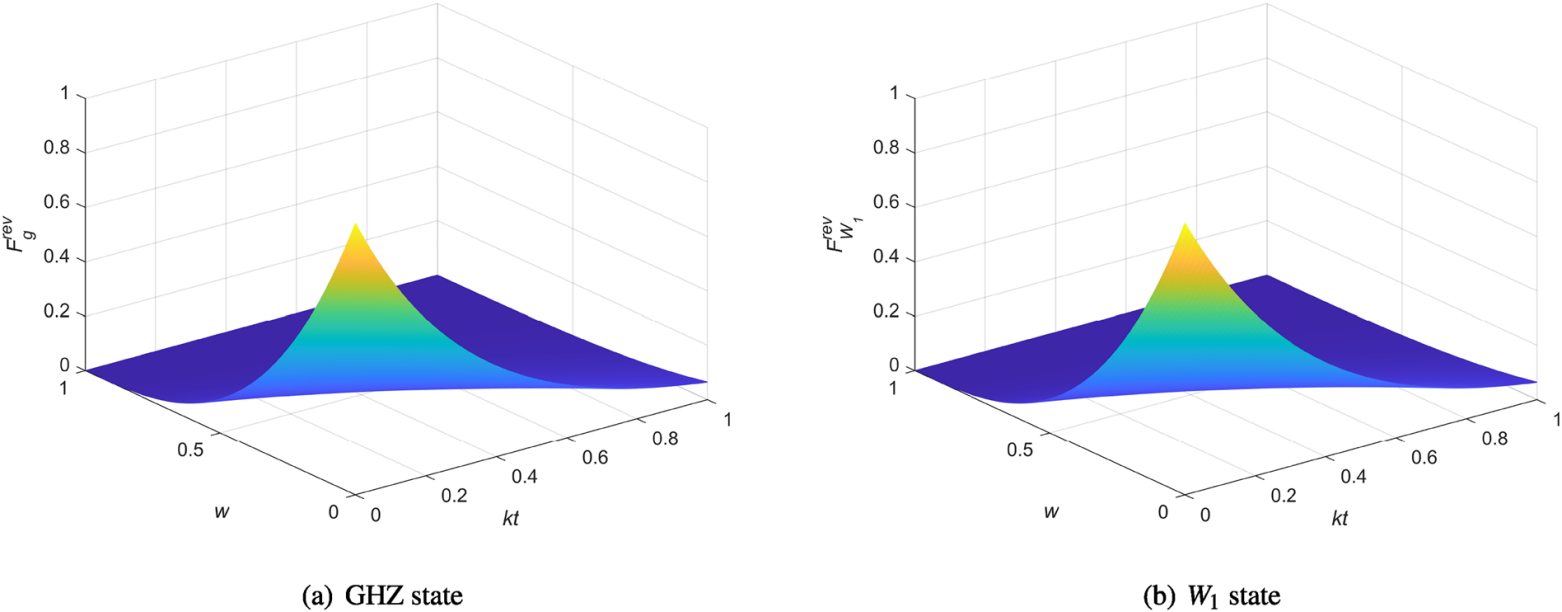
The fidelities of quantum teleportation using three-qubit entangled state with the help of WM and RM under amplitude damping noise.

In previous sections, we considered the fidelities under amplitude damping noise for GHZ and $W_1$ states, respectively. Now, we concentrate on analyzing the fidelities of these two situations with the help of WM and RM. The comparison between GHZ and $W_1$ is given in Fig. 3. Here, we consider a case where all the particles to be sent are affected by the same noise, the same strength of WM and RM. That is, $k_i=k, w_i=w, r_i=r, i=1, 2, 3.$ Moreover, we use the optimal reversing condition $r=w+(1-e^{-kt})(1-w)$^43,44^. And we assume that Alice wants to transmit a state $|\varphi \rangle _a=\frac{1}{\sqrt{2}}(|0\rangle _a+|1\rangle _a)$ to Bob.

Our calculations and simulation results are shown in Fig. 3. The fidelities of quantum teleportation are not only related to the quantum noise parameter, but also affected by the strength of WM when the input state is determined. Plot (a) shows that the fidelity of teleportation with WM and RM by using GHZ state as quantum channel under amplitude damping noise. Obviously, the fidelity of teleportation using GHZ state decreases with the increased strength *w*. It is worth pointing out that the fidelity will tend to 0, when $w>0.5$ or $kt>0.5$, namely, the longer the decoherence time and the stronger the WM, the greater the impact on the fidelity. Plot (b) is the result of $W_1$ state. In this case, the appearance of fidelity is similar to the previous one. Surprisingly, both the teleportation fidelities of GHZ and $W_1$ state are always smaller if the WM and RM are performed in the communication process. It can be inferred that WM and RM cannot improve the efficiency of QT for the given protocol, although it is able to increase other quantum resources of the entangled state with a certain probability.

**Figure 4:**
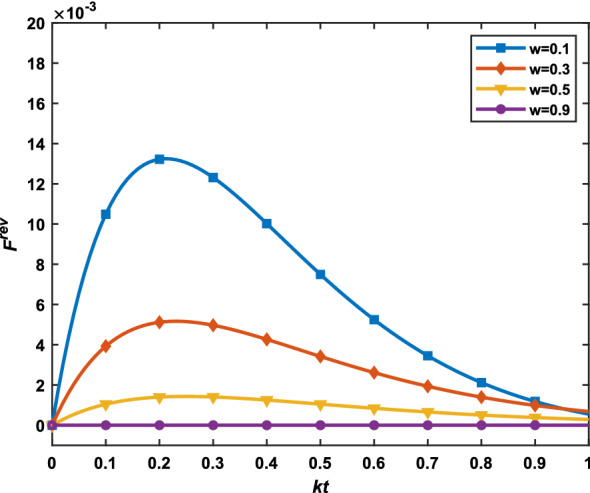
The comparison of fidelities of quantum teleportation between GHZ state and $W_1$ state with the help of WM and RM.

In order to further discuss the influence of GHZ and $W_1$ states as quantum channels on communication efficiency, we subtracted the fidelity of GHZ $(F_{GHZ}^{rev})$ from the fidelity of $W_1$ $(F_{W_1}^{rev})$, which is

33 $$\begin{aligned} F^{rev}=F_{W_1}^{rev}-F_{GHZ}^{rev}, \end{aligned}$$

where $\alpha =\beta =\frac{1}{\sqrt{2}}, k_i=k, w_i=w, r_i=r, i=1, 2, 3,$ and $r=w+(1-e^{-kt})(1-w).$ Figure 4 shows the difference between GHZ and $W_1$ states in their fidelity values under the same condition. When $0<w<1$, the value of $F^{rev}$ decreases with the increase of *w* and it is always greater than or equal to 0, which indicates that $W_1$ can resist more amplitude damping noise than GHZ state at the same time. The result further suggests the WM and its inverse operation have no positive effect on the efficiency of teleportation with GHZ and $W_1$ state under amplitude damping noise.

## Discussion

Here, we discuss what happens if we make minor modifications to the QT protocol. Different from the description of Results-Quantum teleportation with GHZ state and $W_1$ state, we assume that Alice prepares the entangled state. In fact, only particle 3 will be sent to Bob by Alice in this situation. Naturally, one can study the fidelity of teleportation by considering particle 3 through amplitude damping noise. For GHZ state, the fidelity can be directly given on the basis of Eq. (19), which is

34 $$\begin{aligned} \begin{aligned} F_g^A& = \frac{1}{2}\left[ \left( \alpha ^4+\beta ^4\right) \left( 1+e^{\frac{-k_3t}{2}}\right) \right] +2\alpha ^2\beta ^2e^{\frac{-k_3t}{2}}. \end{aligned} \end{aligned}$$

For the case of $W_1$ state, we can obtain the fidelity by Eq. (25), the calculation result is shown as

35 $$\begin{aligned} \begin{aligned} F^A_{W_1}& = \frac{1}{8}\left[ \left( 1+\alpha ^2e^{\frac{-k_1t}{2}}+\beta ^2\right) ^2 +\left( 1+\alpha ^2+\beta ^2e^{\frac{-k_1t}{2}}\right) ^2\right] . \end{aligned} \end{aligned}$$

If we consider that Bob prepares the initial entangled state $|\phi _g\rangle$, then Bob sends qubit-pair (1, 2) to Alice. The fidelities of teleportation also can be obtained by Eqs. (19) and (25) respectively for GHZ state and $W_1$ state, which are

36 $$\begin{aligned} \begin{aligned} F_g^B & = \frac{1}{2}\left[ \left( \alpha ^4+\beta ^4\right) \left( 1+e^{\frac{-k_1t}{2}} +e^{\frac{-k_2t}{2}}\right) \right] +2\alpha ^2\beta ^2e^{\frac{-k_1t}{2}}e^{\frac{-k_2t}{2}}. \end{aligned} \end{aligned}$$

and

37 $$\begin{aligned} \begin{aligned} F^B_{W_1}& = \frac{1}{8}\left[ \left( \alpha ^2+\alpha ^2e^{\frac{-k_2t}{2}} +2\beta ^2e^{\frac{-k_3t}{2}}\right) ^2+ \left( \beta ^2+2\alpha ^2e^{\frac{-k_3t}{2}}+\beta ^2e^{\frac{-k_3t}{2}}\right) ^2\right] . \end{aligned} \end{aligned}$$

**Figure 5:**
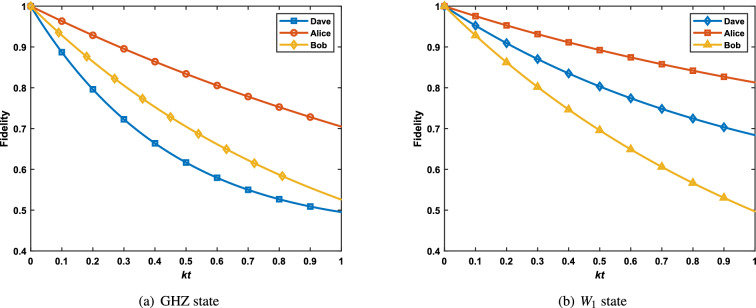
The fidelities of quantum teleportation of the initial states are prepared by Alice, Bob and Dave, respectively.

To compare the fidelities of these two cases with the original case, we performed a simulation of these three cases. Let $k_1=k_2=k_3=k$, $\alpha =\beta =\frac{1}{\sqrt{2}}$ , the numerical results are shown in Fig. 5. Plot (a) shows that the teleportation fidelity when Alice or Bob prepares GHZ state as the initial state is higher than that when Dave prepares the state. The teleportation fidelities about $W_1$ state are shown in plot (b). When Alice prepares the $W_1$ state at her laboratory, the fidelity of QT is more than the situation of preparing state in Dave’s laboratory. While Bob prepares the $W_1$ state, the teleportation fidelity is reduced compared with Dave. Therefore, one can improve teleportation fidelity by selecting the proper preparation laboratory.

In addition, we make comparisons on teleportation fidelities between the modified protocol and original protocol under bit flip, bit-phase flip and phase flip noises, respectively. Similar to the case of amplitude damping noise, one can obtain the fidelities of quantum teleportation under bit flip, bit-phase flip, and phase flip noises by solving the Lindblad master equation, respectively.

Consider the particle to be sent through the noises, which is respectively described by the Lindblad operators $L_{i, \alpha }=\sqrt{k_{i, \alpha }}\sigma _\alpha (\alpha =x, y, z).$ Defining $p_{i, \alpha }=\frac{1}{2}(1+e^{-2k_{i, \alpha }t})$, the evolution described by the Lindblad master equation at time *t* is equivalent to the Kraus operation represented by

38 $$\begin{aligned} E_{i, \alpha }^0=\sqrt{p_{i,\alpha } }I, \quad \quad E_{i, \alpha }^1=\sqrt{1-p_{i,\alpha } }\sigma _\alpha , \end{aligned}$$

where *x*, *y*, *z* correspond to the bit flip, bit-phase flip and phase flip noises.

Suppose that Dave prepare the initial state $|\phi _g\rangle$ and sends particles to Alice and Bob through bit flip, bit-phase flip and phase flip noises respectively. The fidelities of quantum teleportation are

39 $$\begin{aligned} F_{g^x}^D= & {} \frac{1}{8}(D_1^x+D_8^x)+\frac{1}{2}\alpha ^2\beta ^2(D_2^x+D_7^x), \end{aligned}$$

40 $$\begin{aligned} F_{g^y}^D= & {} \frac{1}{8}(D_1^y+4\alpha ^2\beta ^2D_7^y), \end{aligned}$$

and

41 $$\begin{aligned} F_{g^z}^D=\frac{1}{8}D_1^z, \end{aligned}$$

where

42 $$\begin{aligned} \begin{aligned}{}&D_1^\alpha =(1+e^{-2k_{1, \alpha }t})(1+e^{-2k_{2, \alpha }t})(1+e^{-2k_{3, \alpha }t}), \quad D_2^\alpha =(1+e^{-2k_{1, \alpha }t})(1+e^{-2k_{2, \alpha }t})(1-e^{-2k_{3, \alpha }t}),\\&D_3^\alpha =(1+e^{-2k_{1, \alpha }t})(1-e^{-2k_{2, \alpha }t})(1+e^{-2k_{3, \alpha }t}), \quad D_4^\alpha =(1+e^{-2k_{1, \alpha }t})(1-e^{-2k_{2, \alpha }t})(1-e^{-2k_{3, \alpha }t}),\\&D_5^\alpha =(1-e^{-2k_{1, \alpha }t})(1+e^{-2k_{2, \alpha }t})(1+e^{-2k_{3, \alpha }t}), \quad D_6^\alpha =(1-e^{-2k_{1, \alpha }t})(1+e^{-2k_{2, \alpha }t})(1-e^{-2k_{3, \alpha }t}),\\&D_7^\alpha =(1-e^{-2k_{1, \alpha }t})(1-e^{-2k_{2, \alpha }t})(1+e^{-2k_{3, \alpha }t}), \quad D_8^\alpha =(1-e^{-2k_{1, \alpha }t})(1-e^{-2k_{2, \alpha }t})(1-e^{-2k_{3, \alpha }t}).\\ \end{aligned} \end{aligned}$$

If Alice or Bob prepares the initial state $|\phi _g\rangle$, one can obtain the corresponding fidelities by setting $k_{3, \alpha }=0$ or $k_{1, \alpha }=k_{2, \alpha }=0$.

When Dave prepares the initial state, the fidelities of teleportation using $W_1$ state under bit flip, bit-phase flip and phase flip noises are

43 $$\begin{aligned} F_{W_1^x}^D= & {} \frac{1}{8}(D_1^x+\alpha ^2\beta ^2D_8^x) +\frac{1}{4}\alpha ^2\beta ^2(2D_2^x+D_3^x+D_5^x)+\frac{1}{16} \left[ D_4^x+D_6^x+(\alpha ^4+\beta ^4)D_7x\right] , \end{aligned}$$

44 $$\begin{aligned} F_{W_1^y}^D= & {} \frac{1}{8}D_1^y+\frac{1}{16}\left[ D_4^y+D_6^y+(\alpha ^4+\beta ^4)(D_7^y+D_8^y)\right] , \end{aligned}$$

and

45 $$\begin{aligned} F_{W_1^z}^D=\frac{1}{8}(D_1^z+D_8^z)+\frac{1}{16}(\alpha ^4+\beta ^4)(D_3^z+D_4^z+D_5^z+D_6^z). \end{aligned}$$

Let $k_{3, \alpha }=0$ or $k_{1, \alpha }=k_{2, \alpha }=0$, then the fidelities of quantum teleportation of the initial states $W_1$ are prepared by Alice or Bob are calculated.

**Figure 6:**
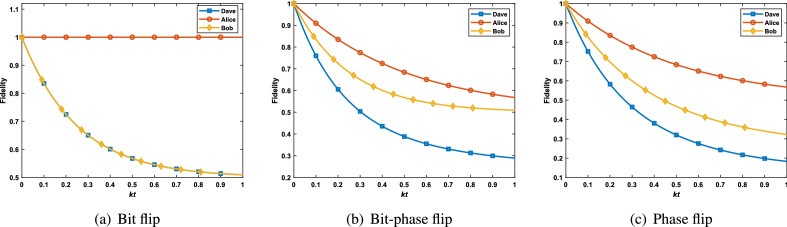
The fidelities of quantum teleportation of GHZ states are prepared by Alice, Bob and Dave, respectively.

**Figure 7:**
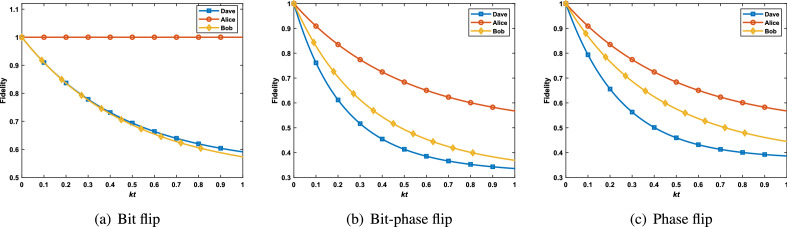
The fidelities of quantum teleportation of $W_1$ states are prepared by Alice, Bob and Dave, respectively.

To demonstrate the effect of bit flip, bit-phase flip and phase flip noises on the the modified protocol and original protocol, we set all parameters $k_{i, \alpha } (i=1, 2, 3, \alpha =x, y, z)$ be equal to *k* and $\alpha =\beta =\frac{1}{\sqrt{2}}$. The simulation results for GHZ state and $W_1$ state are shown in Figs. 6 and 7, respectively. The figures show that when Alice and Bob prepared the initial entangled state, the fidelities of QT are higher than that Dave under all the three types of noises. In particular, the fidelity of QT is not affected by the bit flip noise in the case that the initial state (GHZ state or $W_1$ state) is prepared by Alice. Therefore, choosing a suitable preparation laboratory can improve the fidelity of QT.

## Conclusion

In this work, we study the efficiency of QT with three-qubit entangled state in a noisy environment by solving the Lindblad master equation. The result shows that using $W_1$ for teleportation can resist more amplitude damping noise than GHZ state during the same time. We further consider the efficiency that quantum teleportation with the help of WM and RM. It shows that $W_1$ always performs better than GHZ under the same practical condition. Furthermore, we found that for the GHZ and $W_1$ states used in the given QT protocol, performing WM and its inverse in amplitude damping noise did not improve fidelity. The research presented here shows that WM and its inverse do not necessarily increase the efficiency of teleportation. Finally, we demonstrate that the fidelities of QT protocol under various types of noises are closely related to the participant who prepares the initial state. It indicates that one can improve the fidelity of QT by modifying the protocol to choose the optimal preparation laboratory.

## Methods

### Amplitude damping noise

For the given quantum teleportation, Alice and Bob should share a three-qubit entangled state and the three transmitted qubits always undergo quantum noises during the phase of entanglement distribution. Suppose $\hat{\rho }$ is the entangled state before it is sent to Alice and Bob. For what concerns the present work, we restrict our analysis to the amplitude damping noise, which is an important example of open quantum system evolution^15^. The master equation describing the dynamics of entangled quantum state under noises is given by Eq. (5).

Consider the particle to be sent through the amplitude damping noise, which is described by the Lindblad operator $L_{i, \alpha }=\sqrt{k_{i, -}}\sigma _-^{(i)},$ $$\sigma _-=\left( \begin{array}{cc} 0 &{} 1\\ 0 &{} 0 \\ \end{array} \right)$$, the constant $k_{i, -}$ (or $k_i$) is approximately equals to the inverse of decoherence time. Moreover, the first term of Eq. (5) can be removed when we make a change of variables. We consider that $H_s$ is a time independent Hermitian matrix, then one can assume that $H_s=0$. Accordingly, the equation of motion for $\hat{\rho }$ is easily found to be

46 $$\begin{aligned} \begin{aligned} \frac{\partial \hat{\rho }}{\partial t}& = k_1\left( \sigma _-^{(1)} \hat{\rho }\sigma _+^{(1)}-\frac{1}{2}\sigma _+^{(1)}\sigma _-^{(1)}\hat{\rho } -\frac{1}{2}\hat{\rho }\sigma _+^{(1)}\sigma _-^{(1)}\right) +k_2\left( \sigma _-^{(2)}\hat{\rho }\sigma _+^{(2)}-\frac{1}{2}\sigma _+^{(2)} \sigma _-^{(2)}\hat{\rho }-\frac{1}{2}\hat{\rho }\sigma _+^{(2)}\sigma _-^{(2)}\right) \\ & \quad +k_3\left( \sigma _-^{(3)}\hat{\rho }\sigma _+^{(3)}-\frac{1}{2} \sigma _+^{(3)}\sigma _-^{(3)}\hat{\rho }-\frac{1}{2}\hat{\rho }\sigma _+^{(3)}\sigma _-^{(3)}\right) , \end{aligned} \end{aligned}$$

where $$\sigma _\pm ^{(1)}=\sigma _\pm \otimes I \otimes I, \sigma _\pm ^{(2)}=I\otimes \sigma _\pm \otimes I, \sigma _\pm ^{(3)}=I \otimes I\otimes \sigma _\pm ,$$ and $$\sigma _+=\left( \begin{array}{cc} 0 &{} 0\\ 1 &{} 0 \\ \end{array} \right)$$.

#### GHZ state

We now investigate the protocol of QT by using a generalized GHZ state under amplitude damping noise. For simplicity, one can directly discuss that the entangled state is affected by the amplitude damping noise, which is shown in Eq. (46). Assume that Alice and Bob use a generalized GHZ state to achieve QT, which has the form of

47 $$\begin{aligned} |\phi _g\rangle _{123}=\lambda _0|000\rangle +\lambda _1|111\rangle , \end{aligned}$$

where $\lambda _0, \lambda _1$ are real and satisfy $|\lambda _0|^2+|\lambda _1|^2=1.$ Its density matrix is

48 $$\begin{aligned} \begin{aligned} \hat{\rho }_g(0)=\left( \begin{array}{cccccccc} \lambda _0^2 &{} 0&{}0&{}0&{}0&{}0&{}0&{}\lambda _0\lambda _1\\ 0&{}0&{}0&{}0&{}0&{}0&{}0&{}0 \\ 0&{}0&{}0&{}0&{}0&{}0&{}0&{}0 \\ 0&{}0&{}0&{}0&{}0&{}0&{}0&{}0 \\ 0&{}0&{}0&{}0&{}0&{}0&{}0&{}0 \\ 0&{}0&{}0&{}0&{}0&{}0&{}0&{}0 \\ 0&{}0&{}0&{}0&{}0&{}0&{}0&{}0 \\ \lambda _0\lambda _1&{} 0&{}0&{}0&{}0&{}0&{}0&{} \lambda _1^2 \end{array} \right) . \end{aligned} \end{aligned}$$

The master equation (46) reduces to 8 diagonal equations and 28 off-diagonal equations. One can easily solve these equations with the initial conditions in Eq. (48). The analytic form of a generalized GHZ state evolution under amplitude damping noise is

49 $$\begin{aligned} \hat{\rho }_g(t)=\begin{pmatrix}\rho ^g_{00}(t)&{}\cdots &{}\rho ^g_{07}(t)\\ \vdots &{}\ddots &{}\vdots \\ \rho ^g_{70}(t)&{}\cdots &{}\rho ^g_{77}(t) \end{pmatrix}, \end{aligned}$$

where the elements are all zero except

50 $$\begin{aligned} \left\{ \begin{aligned}&\rho ^g_{00}(t)=1-\lambda _1^2\left[ e^{-t(k_1+k_2+k_3)}+e^{-tk_1}+e^{-tk_2} +e^{-tk_3}-e^{-t(k_1+k_2)}-e^{-t(k_1+k_3)}-e^{-t(k_2+k_3)}\right] ,\\&\rho ^g_{11}(t)=\lambda _1^2\left[ e^{-t(k_1+k_2+k_3)}+e^{-tk_3}-e^{-t(k_1+k_2)}-e^{-t(k_2+k_3)}\right] ,\\&\rho ^g_{22}(t)=\lambda _1^2\left[ e^{-t(k_1+k_2+k_3)}+e^{-tk_2}-e^{-t(k_1+k_2)}-e^{-t(k_2+k_3)}\right] ,\\&\rho ^g_{33}(t)=\lambda _1^2\left[ -e^{-t(k_1+k_2+k_3)}+e^{-t(k_2+k_3)}\right] ,\\&\rho ^g_{44}(t)=\lambda _1^2\left[ e^{-t(k_1+k_2+k_3)}+e^{-tk_1}-e^{-t(k_1+k_2)}-e^{-t(k_1+k_3)}\right] ,\\&\rho ^g_{55}(t)=\lambda _1^2\left[ -e^{-t(k_1+k_2+k_3)}+e^{-t(k_1+k_3)}\right] ,\\&\rho ^g_{66}(t)=\lambda _1^2\left[ -e^{-t(k_1+k_2+k_3)}+e^{-t(k_1+k_2)}\right] ,\\&\rho ^g_{77}(t)=\lambda _1^2\left[ e^{-t(k_1+k_2+k_3)}\right] ,\\&\rho ^g_{07}(t)(\rho ^g_{70}(t))=\lambda _0\lambda _1\left[ e^{-\frac{t}{2}(k_1+k_2+k_3)}\right] .\\ \end{aligned} \right. \end{aligned}$$

On the other hand, defining $p=1-e^{-kt}$ at time *t*, the evolution is equivalent to

51 $$\begin{aligned} \begin{aligned} \hat{\rho }_g(t)&=\varepsilon (\hat{\rho }_g(0)) =\sum _{l=0}^1\sum _{m=0}^1\sum _{k=0}^1E_l(1)E_m(2)E_k(3)\hat{\rho }_g(0)E_l^\dagger (1) E_m^\dagger (2)E_k^\dagger (3) \end{aligned} \end{aligned}$$

where $E_l(1)=E_l\otimes I\otimes I, E_m(2)=I\otimes E_m \otimes I, E_k(3)=I\otimes I \otimes E_k$ and $$E_0=\left( \begin{array}{cc} 1 &{} 0\\ 0&{} \sqrt{1-p} \\ \end{array} \right)$$, $$E_1=\left( \begin{array}{cc} 0 &{} 0\\ 0&{} \sqrt{p} \\ \end{array} \right)$$. Therefore, the evolved state is rewritten as

52 $$\begin{aligned} \begin{aligned} \hat{\rho }_g(t)& = \left( \lambda _0|000\rangle +\lambda _1 e^{\frac{-k_1t}{2}}e^{\frac{-k_2t}{2}} e^{\frac{-k_3t}{2}}|111\rangle \right) \left( \lambda _0\langle 000|+\lambda _1 e^{\frac{-k_1t}{2}}e^{\frac{-k_2t}{2}} e^{\frac{-k_3t}{2}}\langle 111|\right) +\lambda _1^2e^{-k_1t}e^{-k_2t}\bar{e}^{-k_3t}|110\rangle \langle 110|\\ & \quad +\lambda _1^2e^{-k_1t}\bar{e}^{-k_2t}e^{-k_3t}|101 \rangle \langle 101|+\lambda _1^2e^{-k_1t}\bar{e}^{-k_2t} \bar{e}^{-k_3t}|100\rangle \langle 100|+\lambda _1^2\bar{e}^{-k_1t}e^{-k_2t}e^{-k_3t}|011\rangle \langle 011|\\ & \quad +\lambda _1^2\bar{e}^{-k_1t}e^{-k_2t}\bar{e}^{-k_3t}|010 \rangle \langle 010|+\lambda _1^2\bar{e}^{-k_1t}\bar{e}^{-k_2t}e^{-k_3t}|001\rangle \langle 001| +\lambda _1^2\bar{e}^{-k_1t}\bar{e}^{-k_2t}\bar{e}^{-k_3t}|000\rangle \langle 000|. \end{aligned} \end{aligned}$$

where $\bar{e}^{-k_1t}=1-e^{-k_1t}, \bar{e}^{-k_2t}=1-e^{-k_2t}, \bar{e}^{-k_3t}=1-e^{-k_3t}.$ Combined with the analysis above, one can obtain the evolved state $\rho _g(t)$, which describes the whole system under noise. The expression is given by

53 $$\begin{aligned} \begin{aligned} \rho _g(t)& = \rho _{in}\otimes \hat{\rho }_g(t).\\ & = \left( \alpha \lambda _0|0000\rangle +\alpha \lambda _1e^{\frac{-k_1t}{2}}e^{\frac{-k_2t}{2}} e^{\frac{-k_3t}{2}}|0111\rangle +\beta \lambda _0|1000\rangle +\beta \lambda _1e^{\frac{-k_1t}{2}} e^{\frac{-k_2t}{2}}e^{\frac{-k_3t}{2}}|1111\rangle \right) \left( \alpha \lambda _0\langle 0000|+\alpha \lambda _1 e^{\frac{-k_1t}{2}}e^{\frac{-k_2t}{2}}e^{\frac{-k_3t}{2}}\langle 0111|\right. \\ & \quad \left. +\beta \lambda _0\langle 1000|+\beta \lambda _1e^{\frac{-k_1t}{2}}e^{\frac{-k_2t}{2}} e^{\frac{-k_3t}{2}}\langle 1111|\right) +\lambda _1^2e^{-k_1t}e^{-k_2t}\bar{e}^{-k_3t} (\alpha |0110\rangle +\beta |1110\rangle )(\alpha \langle 0110|+\beta \langle 1110|)\\ & \quad +\lambda _1^2e^{-k_1t}\bar{e}^{-k_2t}e^{-k_3t}(\alpha |0101\rangle +\beta |1101\rangle ) (\alpha \langle 0101|+\beta \langle 1101|)+\lambda _1^2e^{-k_1t}\bar{e}^{-k_2t}\bar{e}^{-k_3t} (\alpha |0100\rangle +\beta |1100\rangle )(\alpha \langle 0100|+\beta \langle 1100|)\\ & \quad +\lambda _1^2\bar{e}^{-k_1t}e^{-k_2t}e^{-k_3t}(\alpha |0011\rangle +\beta |1011\rangle ) (\alpha \langle 0011|+\beta \langle 1011|)+\lambda _1^2\bar{e}^{-k_1t}e^{-k_2t}\bar{e}^{-k_3t} (\alpha |0010\rangle +\beta |1010\rangle )(\alpha \langle 0010|+\beta \langle 1010|)\\ & \quad +\lambda _1^2\bar{e}^{-k_1t}\bar{e}^{-k_2t}e^{-k_3t}(\alpha |0001\rangle +\beta |1001\rangle ) (\alpha \langle 0001|+\beta \langle 1001|)+\lambda _1^2\bar{e}^{-k_1t}\bar{e}^{-k_2t}\bar{e}^{-k_3t}(\alpha |0000\rangle +\beta |1000\rangle )(\alpha \langle 0000|+\beta \langle 1000|). \end{aligned} \end{aligned}$$

#### W state

In this part we focus on the evolution of another type of three-qubit state under noise. Considering the W state is

54 $$\begin{aligned} |\phi _w\rangle _{123}=x|001\rangle +y|010\rangle +z|100\rangle , \end{aligned}$$

where *x*, *y*, *z* are real and $x^2+y^2+z^2=1$. The initial state prepared by Dave has the form of

55 $$\begin{aligned} \begin{aligned} \hat{\rho }_w(0)=\left( \begin{array}{cccccccc} 0 &{} 0&{}0&{}0&{}0&{}0&{}0&{}0\\ 0&{}x^2&{}xy&{}0&{}0&{}xz&{}0&{}0 \\ 0&{}xy&{}y^2&{}0&{}0&{}yz&{}0&{}0 \\ 0&{}0&{}0&{}0&{}0&{}0&{}0&{}0 \\ 0&{}0&{}0&{}0&{}0&{}0&{}0&{}0 \\ 0&{}xz&{}yz&{}0&{}0&{}z^2&{}0&{}0 \\ 0&{}0&{}0&{}0&{}0&{}0&{}0&{}0 \\ 0&{} 0&{}0&{}0&{}0&{}0&{}0&{}0 \end{array} \right) . \end{aligned} \end{aligned}$$

Suppose that particles 1, 2, 3 are respectively affected by amplitude damping noise, which is also described by Eq. (46). Solving the master equation associate with new boundary conditions of Eq. (55). One can express $\hat{\rho }_w(t)$ analytically in a form

56 $$\begin{aligned} \hat{\rho }_w(t)=\begin{pmatrix}\rho ^w_{00}(t) &{} 0&{}0&{}0&{}\rho ^w_{04}(t)&{}0&{}0&{}0\\ 0&{}\rho ^w_{11}(t)&{}\rho ^w_{12}(t)&{}0&{}0&{}\rho ^w_{15}(t)&{}0&{}0 \\ 0&{}\rho ^w_{21}(t)&{}\rho ^w_{22}(t)&{}0&{}0&{}\rho ^w_{25}(t)&{}0&{}0 \\ 0&{}0&{}0&{}0&{}0&{}0&{}0&{}0 \\ \rho ^w_{40}(t)&{}0&{}0&{}0&{}\rho ^w_{44}(t)&{}0&{}0&{}0 \\ 0&{}\rho ^w_{51}(t)&{}\rho ^w_{52}(t)&{}0&{}0&{}0&{}0&{}0 \\ 0&{}0&{}0&{}0&{}0&{}0&{}0&{}0 \\ 0&{} 0&{}0&{}0&{}0&{}0&{}0&{}0 \end{pmatrix}, \end{aligned}$$

where

57 $$\begin{aligned} \left\{ \begin{aligned}&\rho ^w_{00}(t)=1-x^2e^{-tk_3}-y^2e^{-tk_2}-z^2e^{-tk_1}, \\&\rho ^w_{11}(t)=x^2e^{-tk_3}, \rho ^w_{22}(t)=y^2e^{-tk_2}, \\&\rho ^w_{44}(t)=z^2e^{-tk_1},\\&\rho ^w_{04}(t)(\rho ^w_{40}(t))=xz\left[ e^{-\frac{t}{2}k_1}-e^{-\frac{t}{2}(k_1+2k_3)}\right] ,\\&\rho ^w_{12}(t)(\rho ^w_{21}(t))=xyze^{-\frac{t}{2}(k_2+k_3)},\\&\rho ^w_{15}(t)(\rho ^w_{51}(t))=xze^{-\frac{t}{2}(k_1+2k_3)},\\&\rho ^w_{25}(t)(\rho ^w_{52}(t))=yze^{-\frac{t}{2}(k_1+k_2+k_3)}.\\ \end{aligned} \right. \end{aligned}$$

Similar to the situation of GHZ state, the evolved state $\hat{\rho }_w(t)$ can be written as

58 $$\begin{aligned} \begin{aligned} \hat{\rho }_g(t)& = (xe^{\frac{-k_3t}{2}}|001\rangle +ye^{\frac{-k_2t}{2}}|010\rangle +ze^{\frac{-k_1t}{2}}|100\rangle )(xe^{\frac{-k_3t}{2}}\langle 001| +y e^{\frac{-k_2t}{2}}\langle 010|+ze^{\frac{-k_1t}{2}}\langle 100|) \\ & \quad +(x^2\bar{e}^{-k_3t}+y^2\bar{e}^{-k_2t}+z^2\bar{e}^{-k_1t})|000\rangle \langle 000|. \end{aligned} \end{aligned}$$

Therefore, the whole quantum system under noise becomes

59 $$\begin{aligned} \begin{aligned} \rho _{W}(t)& = (z\alpha e^{\frac{-k_1t}{2}}|0100\rangle +y\alpha e^{\frac{-k_2t}{2}} |0010\rangle +x\alpha e^{\frac{-k_3t}{2}}|0001\rangle +z\beta e^{\frac{-k_1t}{2}}|1100\rangle \\ & \quad +y\beta e^{\frac{-k_2t}{2}}|1010\rangle +x\beta e^{\frac{-k_3t}{2}}|1001 \rangle )(z\alpha e^{\frac{-k_1t}{2}}\langle 0100|+y\alpha e^{\frac{-k_2t}{2}}\langle 0010|\\ & \quad +x\alpha e^{\frac{-k_3t}{2}}\langle 0001|+z\beta e^{\frac{-k_1t}{2}}\langle 1100|+y\beta e^{\frac{-k_2t}{2}}\langle 1010|+x\beta e^{\frac{-k_3t}{2}}\langle 1001|)\\ & \quad +\left( x^2\bar{e}^{-k_3t}+y^2\bar{e}^{-k_2t}+z^2\bar{e}^{-k_1t}\right) (\alpha |0000\rangle +\beta |1000\rangle )(\alpha \langle 0000|+\beta \langle 1000|). \end{aligned} \end{aligned}$$

## Data Availability

The datasets used and analyzed during the current study are available from the corresponding author upon reasonable request.
